# SLUG promotes prostate cancer cell migration and invasion via CXCR4/CXCL12 axis

**DOI:** 10.1186/1476-4598-10-139

**Published:** 2011-11-10

**Authors:** Berna Uygur, Wen-Shu Wu

**Affiliations:** 1 Program in Biochemistry and Molecular Biology, University of Maine, Orono, Maine 04469, USA; 2 Center for Molecular Medicine, Maine Medical Center Research Institute, Maine Medical Center, Scarborough, Maine 04074, USA; 3 Children's Hospital Oakland Research Institute, Oakland, CA 94609, USA

## Abstract

**Background:**

SLUG is a zinc-finger transcription factor of the Snail/Slug zinc-finger family that plays a role in migration and invasion of tumor cells. Mechanisms by which SLUG promotes migration and invasion in prostate cancers remain elusive.

**Methods:**

Expression level of CXCR4 and CXCL12 was examined by Western blot, RT-PCR, and qPCR analyses. Forced expression of SLUG was mediated by retroviruses, and SLUG and CXCL12 was downregulated by shRNAs-expressing lentiviruses. Migration and invasion of prostate cancer were measured by scratch-wound assay and invasion assay, respectively.

**Research:**

We demonstrated that forced expression of SLUG elevated CXCR4 and CXCL12 expression in human prostate cancer cell lines PC3, DU145, 22RV1, and LNCaP; conversely, reduced expression of SLUG by shRNA downregulated CXCR4 and CXCL12 expression at RNA and protein levels in prostate cancer cells. Furthermore, ectopic expression of SLUG increased MMP9 expression and activity in PC3, 22RV1, and DU-145 cells, and SLUG knockdown by shRNA downregulated MMP9 expression. We showed that CXCL12 is required for SLUG-mediated MMP9 expression in prostate cancer cells. Moreover, we found that migration and invasion of prostate cancer cells was increased by ectopic expression of SLUG and decreased by SLUG knockdown. Notably, knockdown of CXCL12 by shRNA impaired SLUG-mediated migration and invasion in prostate cancer cells. Lastly, our data suggest that CXCL12 and SLUG regulate migration and invasion of prostate cancer cells independent of cell growth.

**Conclusion:**

We provide the first compelling evidence that upregulation of autocrine CXCL12 is a major mechanism underlying SLUG-mediated migration and invasion of prostate cancer cells. Our findings suggest that CXCL12 is a therapeutic target for prostate cancer metastasis.

## Introduction

Prostate cancer is the second leading type of cancer in men in United States. In 2010, new cases of prostate cancer were estimated at 217,730, resulting in 32,050 deaths in [1]. The major cause of death is bone metastasis. Metastasis is a very complicated process during which cancer cells go through a series of steps: (i) cell dissociation from the primary tumor environment, (ii) cell adhesion to the endothelial surface at the target, (iii) cell invasion through the endothelial surface, (iv) cell invasion into new environment, and (v) cell proliferation.

In our previous study, we found that SLUG, a zinc-finger transcription factor, was elevated in mouse prostate tumors and human prostate cancer cell lines [2]. SLUG belongs to the Slug/Snail superfamily [3,4], and it regulates epithelial-mesenchymal transition (EMT) in a variety of cancers [5]. EMT is a dynamic process that promotes cell motility with decreased adhesive ability, and thus is thought to be a major starting point for cancer metastasis [6]. SLUG plays a major role in EMT during embryonic development and metastasis of breast cancers, through partial inhibition of E-cadherin [7,8,3].

In the tumor microenvironment, a complex network of chemokines and receptors affects metastasis. The CXCL12/CXCR4 pathway was originally discovered in the immune system to play an important role in cancer cell metastasis [9-12]. Mice deficient of either CXCR4 or CXCL12 had abnormal development in the central nervous system [13]. CXCL12 belongs to chemokine family of small peptides with 8 to 12 kDA size that control cell activation, differentiation, and trafficking [14,15]. CXCL12 is expressed by several organs: lung, liver, skeletal muscle, brain, heart, kidney, skin, and bone marrow; its secretion is related to tissue damage [16]. The CXCR4/CXCL12 axis can coordinate metastasis of a variety of cancers, such as bladder [17], breast [18], head and neck [19], ovarian [20], renal cell [21], and prostate [22,23]. Interestingly, SLUG is required for transcriptional and functional regulation of CXCL12 during bone tissue remodeling [24].

Although the role of SLUG in cancer metastasis has been documented in other cancers besides prostate cancer, its molecular mechanism remains elusive. In this study, we examined the regulation of the Slug-CXC4R/CXCL12-metastasis triangle in an in vitro cell culture model of human prostate cancer cells. We used gain- and loss-of-function approaches to study (i) how SLUG regulates the CXCR4/CXCL12 axis, and (ii) the functional role of CXCL12 in SLUG-induced migration and invasion of human prostate cancer cell lines. We found that forced expression of SLUG significantly upregulated both CXCL12 and CXCR4 expression and their downstream target MMP9. Knockdown of SLUG decreased CXCL12 and CXCR4 expression in prostate cancer cells. Furthermore, we showed that downregulation of CXCL12/CXCR4 axis via CXCL12 knockdown impaired SLUG-mediated MMP9 expression, migration and invasion. Lastly, we provide evidence that CXCL12 and SLUG regulate migration and invasion of prostate cancer cells independent of cell growth. Our findings suggest that prostate cancer cells can gain invasive characteristics through upregulation of autocrine CXCL12.

## Results

### SLUG upregulated CXCL12 expression in prostate cancer cell lines

**Figure 1:**
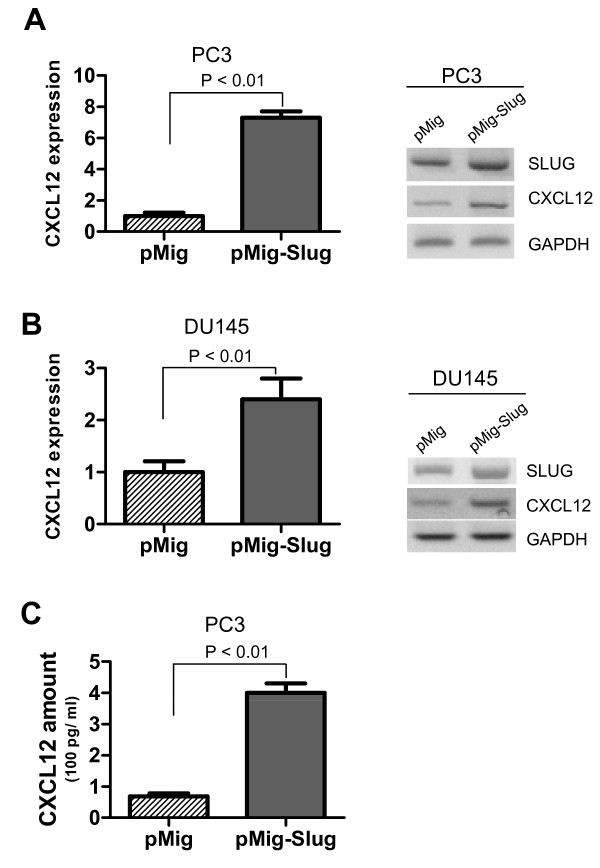
**Analysis of CXCL12 expression in prostate cancer cell lines stably overexpressing SLUG**. (**A**, **B**) Analysis of RNA transcripts of CXCL12 by qPCR (left panel) and RT-PCR (right panel). RNA was extracted from PC3 (A) or DU-145 (B) prostate cancer cell lines infected with pMIGR1-Slug or pMIGR1 (vector) retroviruses, and used to synthesize cDNA. Transcript level of CXCL12 and SLUG was analyzed by qPCR (left panel) and RT-PCR (right panel). GAPDH was included as a loading control. CXCL12 was significantly upregulated by SLUG overexpression in PC3 (A) and DU145 (B). (**C**) ELISA analysis of CXC12 protein in PC3 cell line. Conditioned cell culture medium was collected from the cell culture environment of PC3 cells infected with pMig (vector) or pMig-Slug retroviruses and centrifuged to remove cell lysates, and then used for ELISA.

CXCL12 expression was significantly higher in human prostate cancer tissue than hyperplastic prostate tissues [25], suggesting that CXCL12 has an autocrine regulatory role via its receptor CXCR4 in the regulation of prostate cancer cell migration, invasion, and metastasis [26]. Slug is a zinc-finger transcription factor and its overexpression promotes migration, invasion, and metastasis of various cancer cells [4]. To determine whether CXCL12/CXCR4 axis plays a role in SLUG-mediated migration and invasion of prostate cancer cells in vitro, we first tested if forced expression of SLUG increases CXCL12 expression. We infected PC3 cells and DU145 cells with retroviruses expressing SLUG (pMig-Slug) or control retroviruses (pMig). By qPCR and RT-PCR analysis, we found that CXCL12 transcription level was 7-fold higher in PC3 cell line overexpressing SLUG versus vector (Figure 1A). In addition, we analyzed CXCL12 expression in established DU145 stable cell lines overexpressing SLUG or vector (Figure 1B), and found that its expression was significantly upregulated by SLUG. Using ELISA, we also measured the protein level of CXCL12 in culture medium of PC3 stable cell lines. Our data showed that the CXCL12 protein level was 5-fold higher in PC3 cells stably expressing pMig-Slug versus the pMig vector control (Figure 1C).

### Knockdown of SLUG reduced CXCL12 expression in prostate cancer cells

**Figure 2:**
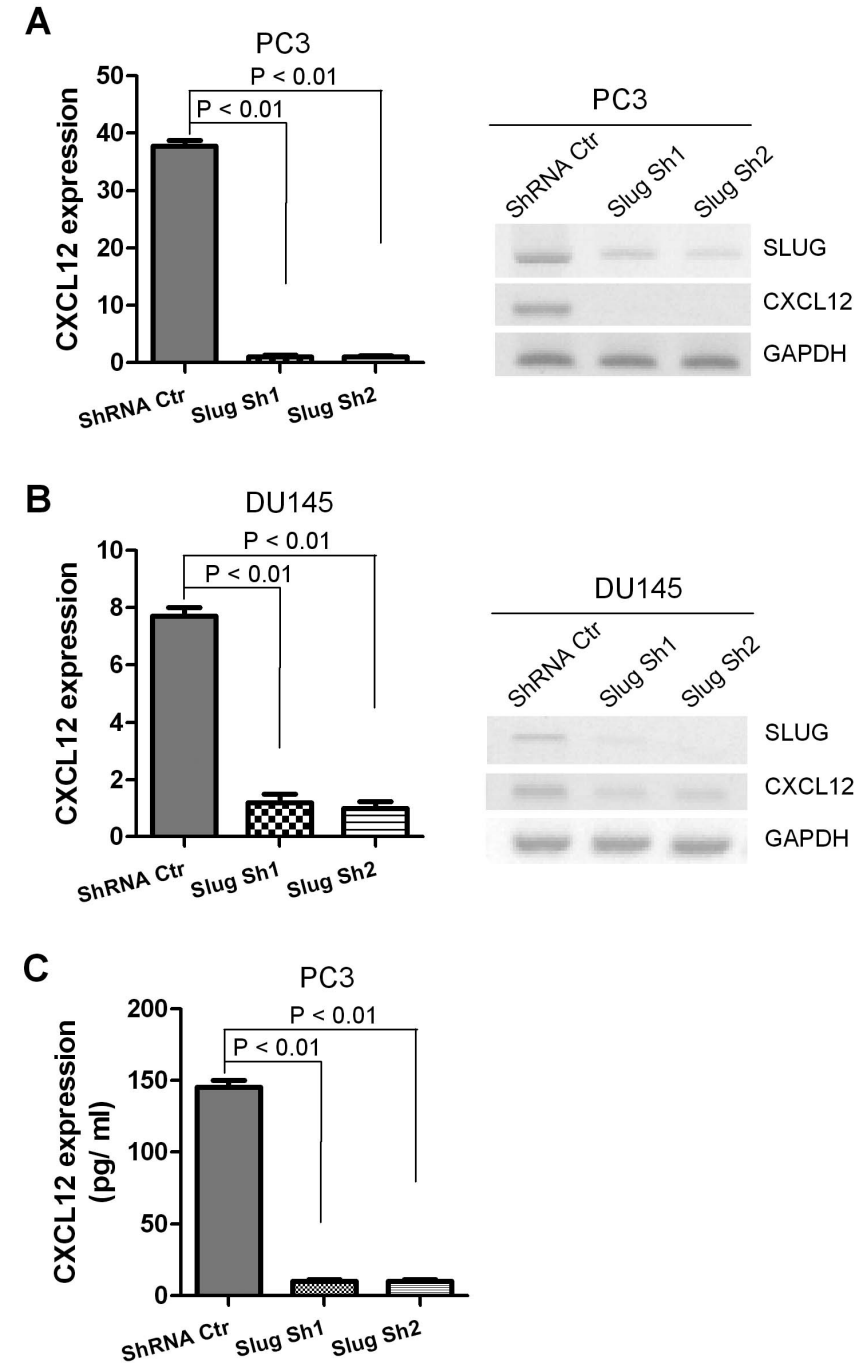
**Examination of CXCL12 RNA transcript levels and protein expression of CXCL12 in prostate cancer cell lines in which SLUG was knocked down by shRNA**. (**A, B**) RNA transcript analysis of CXCL12. RNA was extracted from PC3 and DU-145 prostate cancer cell lines stably expressing Slug shRNA (Sh1 and Sh2) or control shRNA (ShRNA Ctr), and subjected to cDNA synthesis. RNA transcript level of CXCL12 and SLUG in these cell lines was analyzed by qPCR (left panel) and RT-PCR (right panel). CXCL12 expression was significantly downregulated in PC3 and DU145 cells expressing Slug shRNAs at RNA transcript level. (**C**) ELISA analysis of CXC12 protein level in PC3 cells expressing Slug shRNAs. Conditioned cell culture medium was collected from cell culture environments of PC3 cells infected with lentiviruses expressing control shRNA (ShRNA Ctr) and Slug-specific shRNA (Slug Sh1 and Slug Sh2), and then analyzed by ELISA. CXCL12 protein level was great significantly downregulated in PC3 cells expressing Slug shRNA.

In addition to gain-of-function studies, we used a loss-of-function approach to assess the effects of Slug knockdown on CXCL12 expression. We established three stable cell lines in PC3 and DU145 by infecting lentiviruses-expressing control shRNA (Ctr) or small hairpin RNA (shRNA) targeting the human *SLUG *gene (shRNA Sh1, Sh2), followed by selection with puromycin. As we expected, Slug RNA level expression was significantly reduced by two independent SLUG shRNAs (Sh1, Sh2) in PC3 (Figure 2A) and DU145 (Figure 2B), as compared with control shRNA (non-target scramble shRNA). Consistent with Figure 1, our data showed that CXCL12 expression was dramatically downregulated in PC3 and DU145 cell lines harboring SLUG shRNAs versus those carrying control shRNA (Figure 2A, B). Moreover, we measured CXCL12 protein expression in culture medium of these stable cell lines and found that CXCL12 protein concentration was significantly lower in PC3 cells expressing SLUG-specific shRNA versus control shRNA (Figure 2C).

We used gain- and loss-of-function approaches to demonstrate that SLUG is a positive regulator of CXCL12 in prostate cancer cells.

### CXCR4 is a target of SLUG in prostate cancer cell lines

**Figure 3:**
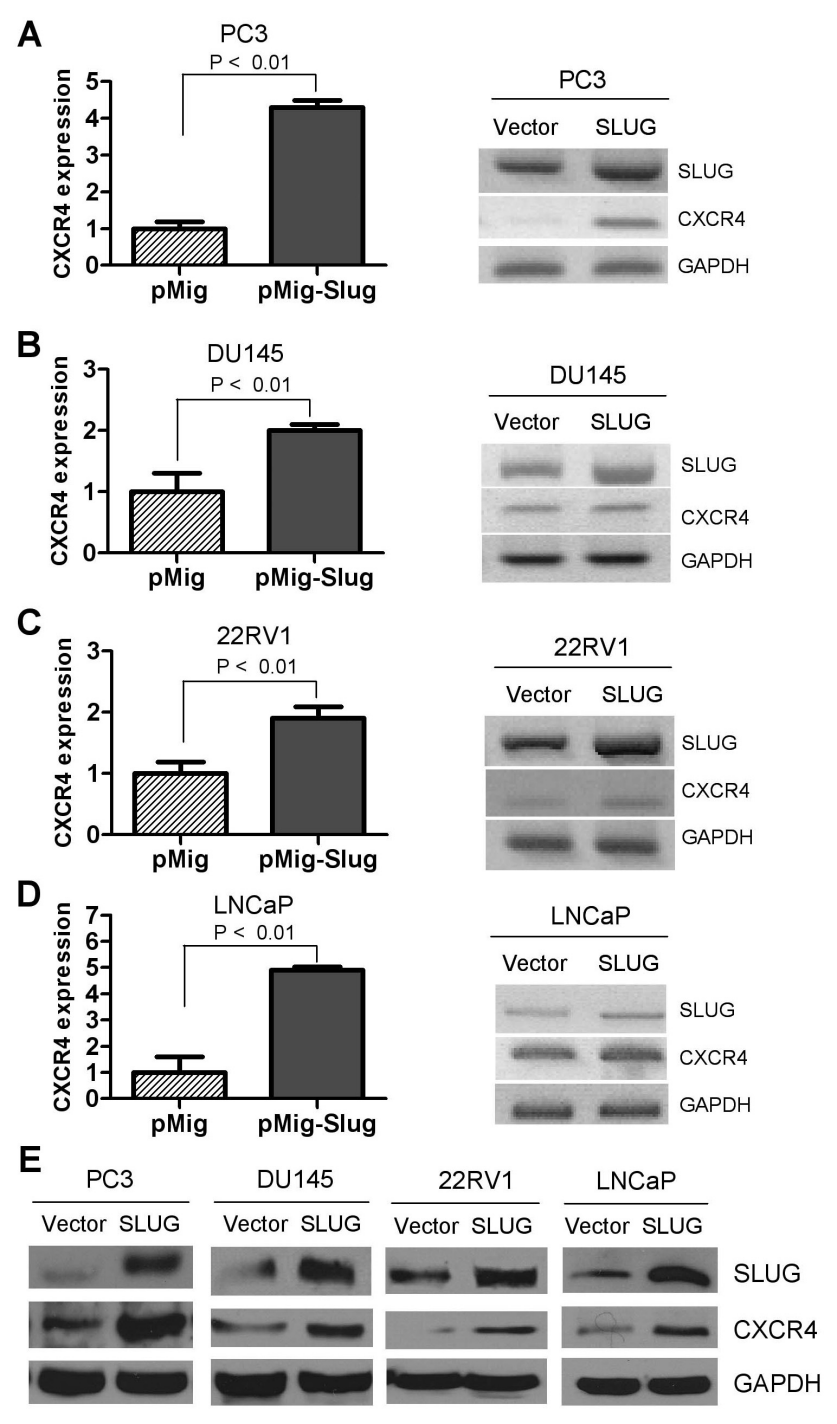
**Analysis of CXCR4 expression in prostate cancer cell lines stably overexpressing SLUG**. (**A**-**D**) qPCR and RT-PCR analysis of RNA transcripts of CXCR4 expression. RNA was extracted from PC3 (**A**), DU145 (**B**), 22RV1 (**C**), and LNCaP (**D**) cell lines stably carrying pMIGR1-Slug or vector control (pMig), and subjected to cDNA synthesis. The transcript level of CXCR4 was analyzed by qPCR (left panel) and RT-PCR (right panel). GAPDH was included as a control. CXCR4 was highly expressed in PC3, DU145, 22RV1, and LNCaP cell lines overexpressing SLUG. (**E**) Western Blot analysis of CXCR4 expression in PC3, DU145, 22RV1, and LNCaP cell lines stably carrying pMig-Slug or pMig (vector). GAPDH was included as a loading control.

CXCR-4 is an alpha-chemokine receptor specific for CXCL12 (also called stromal-derived-factor-1 or SDF-1), a molecule endowed with potent chemotactic activity for lymphocytes and tumor cells. It has been reported that CXCR4 is expressed in prostate cancer cells but not in immortalized prostate epithelial cells [27,28]. In our previous study, we found that SLUG protein expression is elevated in human prostate cancer cell lines [2]. To investigate whether SLUG can also regulate CXCR4 expression in prostate cancer cell lines, we infected four prostate cancer cell lines with retrovirus expressing SLUG (pMig-Slug) or control retroviruses (pMig). We examined CXCR4 expression of both at the transcriptional level and protein level by RT-PCR and qPCR and Western Blot analysis, respectively. Our data showed that forced expression of SLUG significantly increased CXCR4 expression at the transcription level in PC3 (Figure 3A), DU145 (Figure 3B), 22RV1 (Figure 3C), and LNCaP cell lines (Figure 3C), respectively. In addition, we examined the protein level of CXCR4 in these stable cell lines. Consistent with the qPCR and RT-PCR data (Figure 3A-D), Western blot analysis confirmed that forced expression of SLUG increased CXCR4 protein expression in these four prostate cancer cell lines (Figure 3E). In addition, flow cytometric analysis indicated that CXCR4 expression is higher on surface of LNCaP cells stably carrying pMig-Slug versus pMig vector control (Additional file 1, Figure S1).

**Figure 4:**
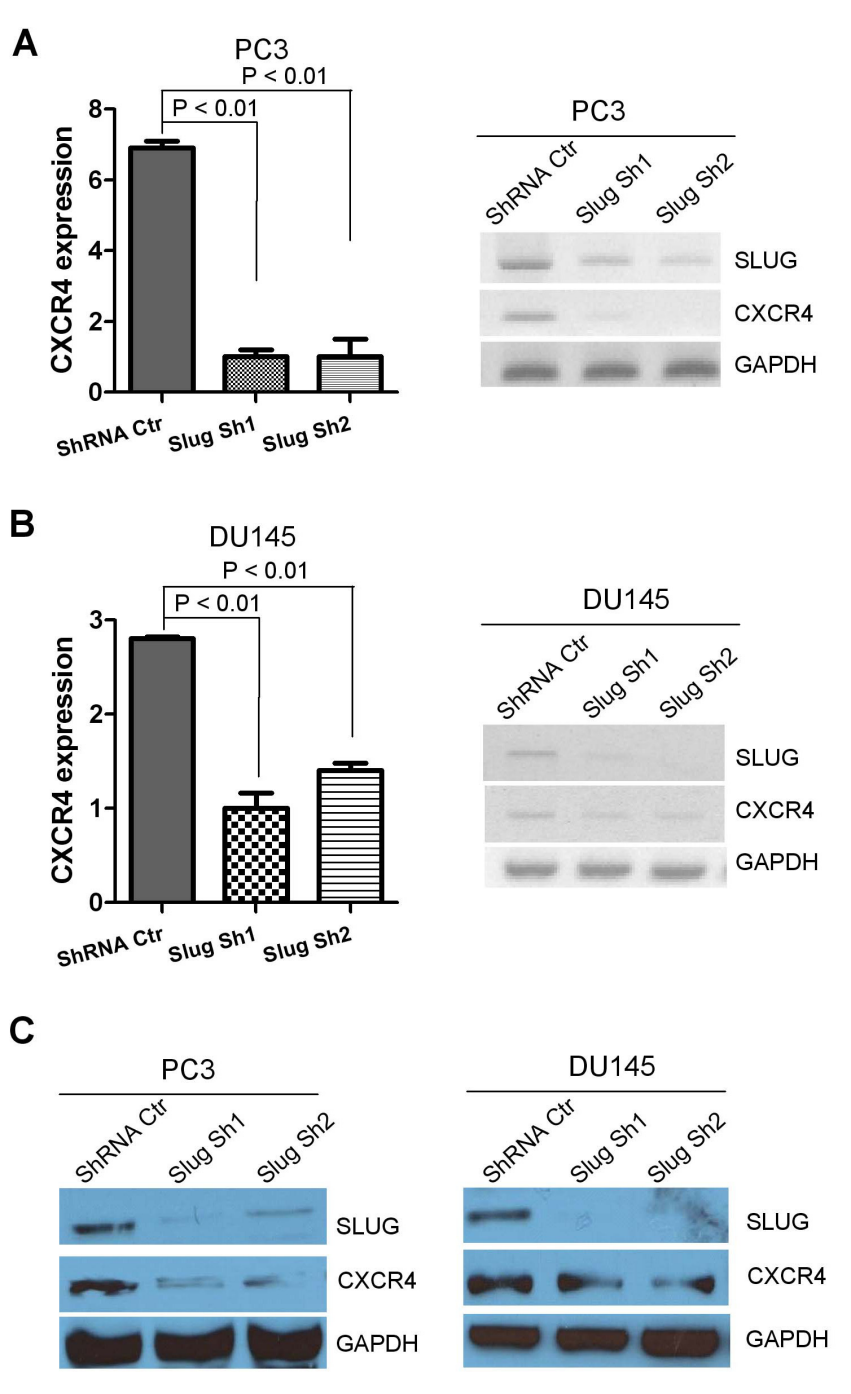
**Examination of CXCR4 expression in prostate cancer cell lines expressing SLUG shRNAs**. (**A**, **B**) Analysis of CXCR4 RNA transcripts by qPCR (left panel) and RT-PCR (right panel). RNA was extracted from PC3 and DU-145 prostate cancer cell lines stably expressing Slug shRNA (Sh1, Sh2) or shRNA control (ShRNA Ctr). All RNA was extracted from these cells four days after infection, and subjected to cDNA synthesis. The CXCR4 and SLUG transcripts were analyzed by qPCR or RT-PCR, or both. GAPDH was included as a control. CXCR4 was significantly downregulated in human prostate cancer cell lines harboring Slug-specific shRNAs. (**C**) Western Blot analysis of CXCR4 protein level in PC3 (left panel) and DU-145 (right panel) lines stably carrying Slug shRNA or control. All protein was extracted and analyzed by Western blot analysis using anti-Slug, anti-CXCR4, anti-gapdh antibodies (loading control).

Next, we asked if endogenous SLUG is required for CXCR4 expression in prostate cancer cell lines. To do so, we confirmed knockdown of SLUG by two independent shRNA (Sh1, Sh2) in both the PC3 and DU145 cell lines (Figure 4A, B). We examined CXCR4 expression in both of these stable cell lines. Our data revealed that SLUG knockdown significantly downregulates CXCR4 expression at the transcriptional level in both PC3 (Figure 4A) and DU145 (Figure 4B) cell lines, by qPCR (Figure 4A, B, left panels) and RT-PCR (Figure 4A, B, right panels) analyses. Furthermore, we analyzed protein expression of CXCR4 in these stable cell lines and found that CXCR4 protein was significantly reduced in PC3 (Figure 4C, left panel) and DU145 (Figure 4C, right panel) when SLUG was knocked down by two independent shRNAs. These data, together with Figures 1, 2 and 3, demonstrated that SLUG upregulates CXCR4 and CXCL12 gene expression in human prostate cancer cells.

### SLUG positively regulates CXCR4/CXCL12 downstream target MMP9 in prostate cancer cells

**Figure 5:**
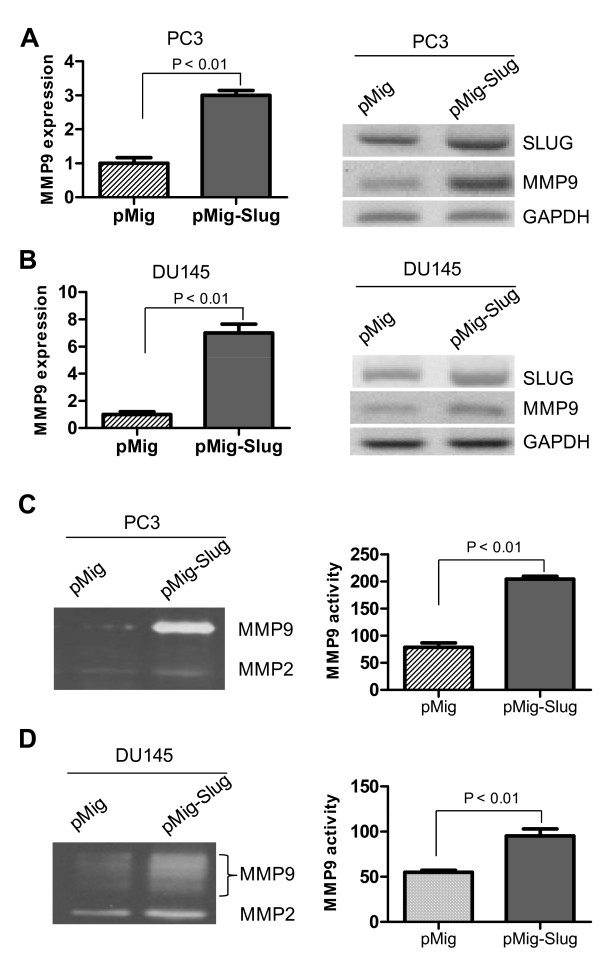
**Overexpression of Slug positively regulates MMP9 expression in prostate cancer cell lines**. (**A**, **B**) Analysis of MMP9 RNA transcripts in prostate cancer cell lines overexpressing SLUG by qPCR (left panel) and RT-PCR (right panel). RNA was extracted from PC3 and DU-145 cell lines stably carrying pMig-Slug or pMig (vector) and then subjected to cDNA synthesis. Transcript level of MMP9 was analyzed by qPCR and RT-PCR. GAPDH was included as a control. MMP9 was highly expressed in PC3 and DU145 overexpressing SLUG. (**C, D**) Zymographic analysis of MMP9 activity in prostate cancer cells overexpressing SLUG. Culture medium were collected from PC3 (**C**) and DU145 (**D**) cell lines stably carrying pMig or pMig-Slug and then assayed for MMP9 activity by gelatin-zymography. The left and right panels are gel image and relative MMP9 activity based on density of bands, respectively.

**Figure 6:**
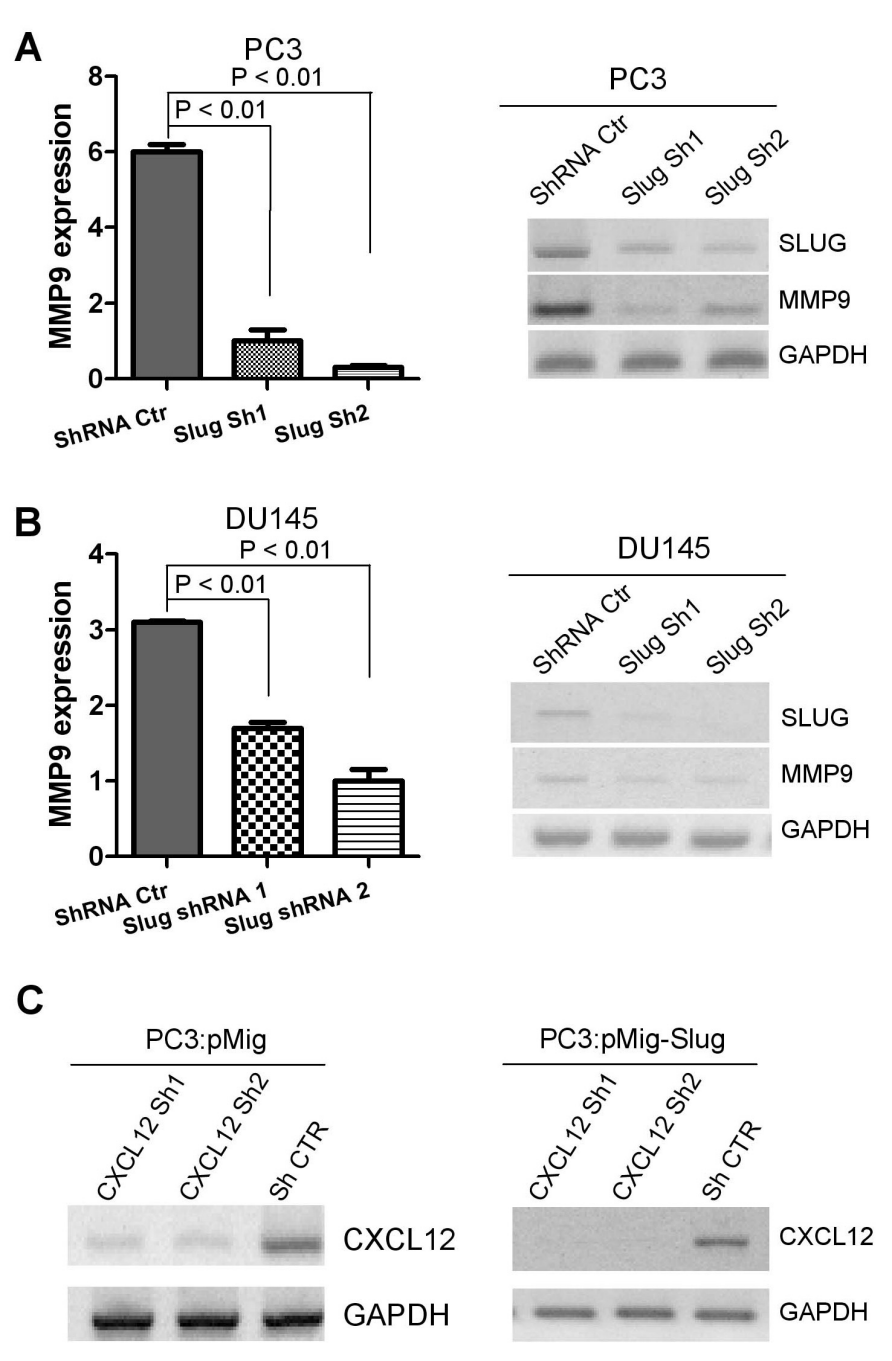
**MMP9 and CXCL12 expression in prostate cancer cells expressing shRNAs targeting SLUG or CXCL12 mRNA**. (**A, B**) qPCR (left panel) and RT-PCR (right panel) analysis of MMP9 RNA transcripts in prostate cancer cell lines harboring SLUG shRNAs. RNA was extracted from PC3 and DU145 cell lines stably expressing SLUG shRNA (Sh1, Sh2) or control shRNA (Ctr), and subjected to cDNA synthesis. Transcript level of MMP9 in these cell lines was analyzed by qPCR (left panel) and RT-PCR (right panel). GAPDH was included as a control. MMP9 was significantly downregulated in PC3 and DU145 cell lines carrying Slug shRNAs. (**C**) Identification of CXCL12-specific shRNAs. PC3-pMig and PC3-pMig-Slug stable cells lines were infected with lentiviruses expressing control shRNA (sh Ctr) and CXCL12 shRNAs (Sh1, Sh2), and followed by puromycin selection. Total RNA was extracted from these stable cell lines and CXCL12 expression was analyzed by RT-PCR.

Our data suggest that SLUG could positively regulate the CXCL12/CXCR4 signaling in prostate cancer cells, leading to cancer migration and invasion. MMP9 belongs to the matrix metalloproteinase family [29] and is a target of the CXCL12/CXCR4 signaling in cancer cells, including prostate cancer [30]. Therefore, we decided to determine whether or not MMP9 is also positively regulated by SLUG in prostate cancer cells. To address this question, we first examined MMP9 gene expression in prostate cancer cells that stably overexpress SLUG gene by qPCR (Figure 5A, B, left panels) and RT-PCR (Figure 5A, B, right panels). Our data showed that MMP9 expression was significantly higher in PC3 (Figure 5A) and DU145 (Figure 5B) stable cell lines overexpressing SLUG than in cells carrying pMig vector only. Next, we examined MMP9 activity in SLUG-overexpressing prostate cancer cell lines by gelatin-zymography. In agreement with Figure 5A, B, MMP9 activity was significantly elevated by SLUG overexpression in PC3 (Figure 5C) and DU145 (Figure 5D) cell lines. Consistently, when SLUG was knocked down by two independent specific shRNAs in PC3 (Figure 6A) and DU145 (Figure 6B), MMP9 expression was dramatically decreased in these cells. Together, our findings indicate that Slug positively regulates MMP9 expression, possibly via CXCR4/CXCL12 pathway in prostate cancer cells.

### CXCL12 is required for SLUG-mediated MMP9 expression and migration of prostate cancer cells

**Figure 7:**
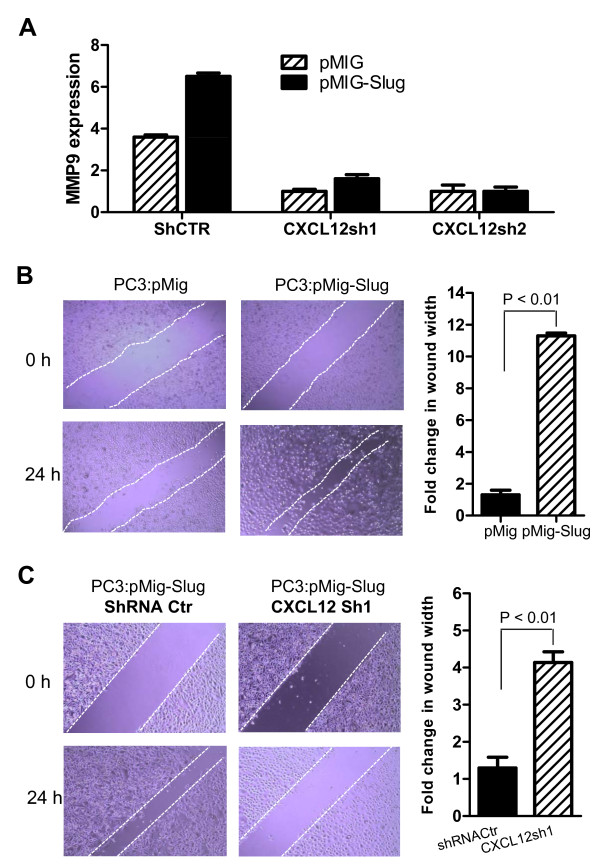
**Knockdown of CXCL12 impaired SLUG-mediated MMP9 expression and prostate cancer cell migration**. (**A**) qPCR analysis of MMP9 expression in PC3 cells overexpressing SLUG and CXCL12 shRNAs. PC3 stable cell lines overexpressing SLUG (or vector) and control shRNA (shCtr) or CXCL12 shRNA (CXCL12sh1 and CXCL12sh2) were proceeded to RNA extraction and cDNA synthesis. MMP9 transcript in these cell lines was analyzed by qPCR. (**B**) Cell migration assay in PC3 cells stably overexpressing SLUG. PC3 cells expressing pMig-Slug or pMig (vector) were seeded in 12-well plates (15 × 10^4 ^cells per well). After cells formed a confluent monolayer, scratches were performed using a 100 μl tip. Twenty-four hours after scratching, the cells were examined for closure of scratch under the microscope and images were captured. Quantification of cell migration was done by measuring the distance between 4 random points within the wound edge in three replicate experiments. (**C**) Cell migration assay in PC3 cells stably overexpressing SLUG and CXCL12 shRNAs. PC3 stable cell lines overexpressing SLUG were infected with control shRNA or CXCL12 shRNA lentiviruses, as indicated. Closure of scratch was examined under the microscope 24 hr after scratching. Quantification of cell migration was done by measuring the distance between 4 random points within the wound edge. Slug-mediated cell migration was diminished in PC3 cells expressing CXCL12 shRNA.

Although our data thus far indicate that both CXCL12 and CXCR4 are positively regulated by SLUG, it remains to be determined if the CXCL12/CXCR4 is a mediator of SLUG-induced MMP9 expression. To address this question, we infected PC3 cell lines overexpressing SLUG or vector with control shRNA or CXCL12 shRNA (Sh1, Sh2)-expressing lentiviruses, and then confirmed efficiency of these shRNAs to knockdown CXCL12 by RT-PCR (Figure 6C). Next, we examined expression of MMP9 in these PC3 stable cell lines by qPCR (Figure 7A) and RT-PCR (Additional file 1, Figure S2). Our data showed that MMP9 expression is significantly higher in PC3 cells co-expressing SLUG and control shRNA, but is not evident in PC3 cells co-expressing SLUG and CXCL12-specific shRNAs (Sh1 and Sh2). These data indicated that CXCL12 is required for SLUG-mediated MMP9 expression in prostate cancer cells.

Furthermore, we performed a scratch-wound assay in the confluent monolayer of cultured stable cell lines. Consistent with published reports [31], our data showed that overexpression of SLUG exhibited a higher scratch closure rate than the controls in metastatic PC-3 cells (Figure 7B) and in non-metastatic 22RV1 cell lines (Additional file 1, Figure S3). Interestingly, SLUG-expressing stable cell lines harboring CXCL12 shRNA showed an impaired scratch closure, compared with the control stable cell line expressing SLUG and control shRNA (Figure 7C). These data indicate that CXCL12 is required for SLUG-mediated MMP9 expression and migration of prostate cancer cells.

### CXCL12 is essential for SLUG-mediated invasion of prostate cancer cells

**Figure 8:**
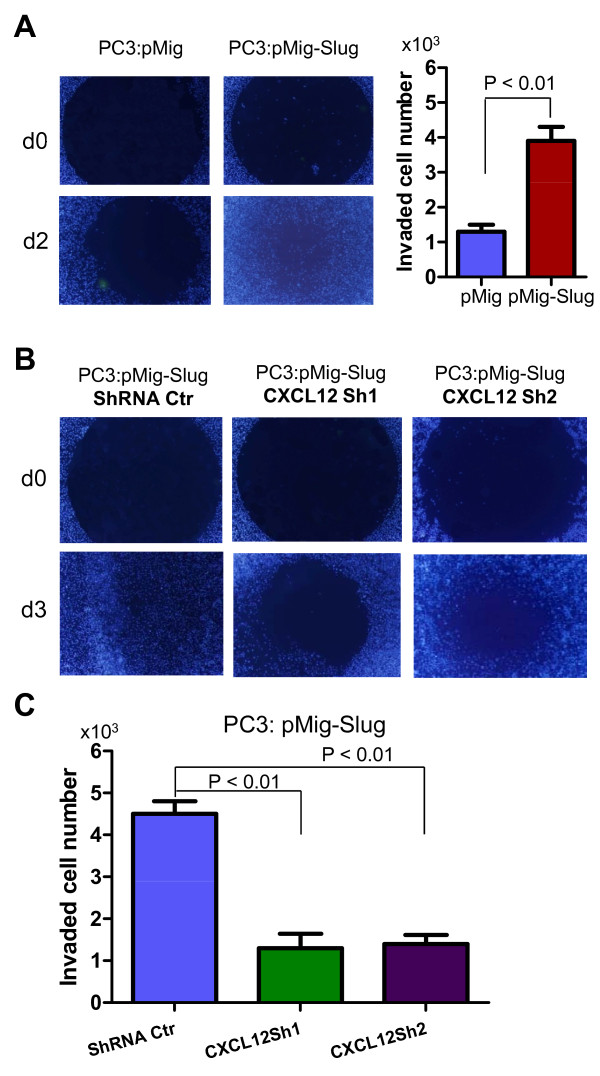
**CXCL12 is required for Slug-mediated prostate cancer cell invasion**. (**A**) Invasion assay of PC3 cells expressing pMig-Slug, or pMig (vector). Briefly, cells were seeded at 6 × 10^4 ^cells per well into 96-well plate from the Oris™ Cell Invasion Assay Kit, and incubated for ~16 hr at 37°C. Collagen I overlay was added to create a 3-D ECM environment for invasion. Cells were allowed to invade for 48 hr, and then stained with DAPI before images were captured (left panel). Total number of invaded cells were manually accounted (right panel). (**B, C**) Invasion assay of PC3 cells overexpressing SLUG and CXCL12 shRNAs. PC3 cell line overexpressing SLUG (pMig-Slug) and control shRNA (Ctr) or CXCL12 shRNA (Sh1, Sh2) were generated as shown in Figure 6C, and their invasion ability was examined as in (A), based on relative numbers of invaded cells (**C**).

Metastasis is characterized by the ability of cancer cells to invade adjacent tissue, and is regulated by multiple signaling pathways, including the CXCL12/CXCR4 axis. Because our data show that SLUG positively regulated both CXCL12 and CXCR4; therefore, we assessed the role of CXCL12 in SLUG-mediated prostate cancer invasion. First, we examined the ability of SLUG to promote prostate cancer invasion by the Oris™ Cell Invasion Assay, which can quantity and image cells invading through an extracellular matrix (ECM). Figure 8A demonstrates overexpression of SLUG increased invasion of PC3 cells. Second, we infected SLUG-expressing PC3 cells with lentiviruses harboring CXCL12 shRNA (Sh1, Sh2) or control shRNA (Ctr). As shown in Figure 8B and 8C, PC3 cell line stably expressing SLUG and shRNA Ctr (Figure 8B, left panel) had a higher invasive ability than the other two stable cell lines co-expressing SLUG and CXCL12 shRNAs (Figure 8B, middle and right panels). Thus, our data indicated that CXCL12 is critical for SLUG-mediated invasion of prostate cancer cells.

### CXCL12 and SLUG regulate migration and invasion of prostate cancer cells independent of cell growth

Because CXCL12 shRNAs relieve SLUG-mediated migration and invasion of prostate cancer cells (Figure 7, 8), we asked whether or not cell proliferation plays a role in these processes. First, we assessed if knockdown of CXCL12 by shRNAs affects cell growth of PC3 cell lines. To do so, we infected PC3 cells with retroviruses expressing shRNA Ctr and two CXCL12 shRNAs (Sh1 and Sh2), respectively. We confirmed efficiency of CXCL12 knockdown by RT-PCR after drug selection (Figure 6C), and then carefully monitored growth of these PC3 stable cell lines by measuring cell numbers of viable cells at each time point. Our data showed that PC3 cell lines expressing shRNA Ctr or two CXCL12 shRNAs (Sh1 and Sh2) had a similar cell proliferation rate (Additional file 1, Figure S4A).

Next, we examined the effects of SLUG overexpression and CXCL12 knowdown on cell growth of PC3 cells in cell culture. As shown in Figure S4B (Additional file 1), the PC3 cell lines expressing SLUG showed a lower proliferation rate than PC3 cell lines with vector, regardless of CXCL12 knockdown. Although CXCL12 shRNAs had no effect on PC3 cell growth (Additional file 1, Figure S4A), CXCL12 knockdown further inhibited growth of PC3 cells overexpressing SLUG (Additional file 1, Figure S4B). Therefore, it is unlikely that CXCL12 knockdown impaired SLUG-mediated migration and invasion of prostate cancer cells by promoting cell growth. Our data suggest that migration and invasion of prostate cancer cells are independent of cell growth.

## Discussion

Metastasis is the spread of a disease from one organ or tissue to another non-adjacent organ or tissue; and thus, it is regulated by numerous signaling pathways in both the cancer cells and microenvironment. CXCR4/CXCL12 axis plays role in cancer cell metastasis and proliferation; the importance of the CXC4/CXCL12 axis may differ in different types of cancer cells, due to their discrete expression. For example, CXCR4 expression is lower in gastrointestinal tumors than breast cancer [32]. Overexpression of CXCR4 in prostate cancer cells accelerated prostate tumor metastasis, prostate tumor vascularization, and tumor growth in vivo [33]. CXCL12 stimulates chemotaxis of metastatic prostate cancer cells expressing a high level of CXCR4 and accelerates their migration [34]. Conversely, blockade of CXCR4/CXCL12 interaction in prostate cancer cells via CXCR4 knockdown significantly inhibits bone metastasis in vivo [35]. Androgens promote migration of prostate cancer cells via KLF5-mediated upregulation of CXCR4 expression [36].

In this study, we used gain- and loss-of-function approaches to determine that SLUG positively regulated both CXCL12 and CXCR4 at the RNA and protein level. Because SLUG is a zinc-finger transcription factor and mainly functions as a transcription repressor when it is tethered to promoters of target genes [4,7], we therefore assumed that SLUG regulates CXCL12 and CXCR4 in an indirect manner, i.e., by suppressing expression of one or more inhibitors of these two molecules. It was recently reported that MiR-886-3p directly targets CXCL12 and decreases its expression [37]. In future studies, we will examine if SLUG directly downregulates MiR-886-3p in prostate cancer cells. Interestingly, CXCL12 can increase the RNA and protein level of the CXCR4 receptor in basal cell carcinoma and PC3 cells [38,39]. Therefore, it is possible that SLUG upregulates CXCR4 in a CXCL12-dependent manner. It has been heavily documented that CXCL12 is expressed in the bone microenvironment and creates migration and invasion paths for the tumor cells with CXCR4 expression [40]. Our current findings indicate that CXCL12 is expressed in prostate cancer cells and was induced by SLUG. Notably, it was recently shown that Slug is required for transcriptional and functional regulation of CXCL12 during the remodeling of bone tissue [24].

Elevated SLUG expression in tumors is correlated with tumor metastasis in many types of tumors [41,25,42], and forced expression of SLUG promotes metastasis of breast cancer in mouse models through partial inhibition of E-cadherin [43]. In this study, we found that SLUG overexpression upregulated endogenous CXCL12 and increased prostate cancer cell migration and invasion, but reduced adhesion (data nor shown). In contrast, knockdown of endogenous CXCL12 expression impaired SLUG-mediated MMP9 expression, and migration and invasion in PC3 cells. Thus, our new findings that CXCL12/CXCR4 is a mediator of SLUG-induced migration and invasion of prostate cancer cells provide insight into the molecular mechanisms by which SLUG promotes tumor cell metastasis in vivo. Neutralizing CXCL12 with specific antibodies in NOD/SCID mice resulted in reduced metastasis to the lungs, adrenal glands, and liver [21]. Therefore, it would be worthwhile to use mouse models to test whether CXCL12 is a key mediator of SLUG-induced metastasis of prostate cancer in vivo.

It has been suggested that CXCL12 promotes tumor invasion by inducing MMP9 [44], which degrades extracellular matrix components. MMP9 is expressed and secreted from both prostate cancer cells and their microenvironment [30,45]. In addition, high expression of SLUG and MMP9 is found in pancreatic cancer tissues [25]. It remains to be determined whether MMP9 is upregulated by SLUG. Here, we showed that SLUG upregulated both CXCL12 and its downstream target MMP9 expression, and that MMP9 is regulated by SLUG through CXCL12. In the future, it needs to be determined if MMP9 is critical for SLUG-induced invasion of prostate cancer cells.

Overall, our data indicate that CXCL12 is a key mediator for SLUG-induced migration and invasion of human prostate cancer cell lines in vitro; thereby suggesting that autocrine CXCL12 is an important factor for tumor metastasis.

## Conclusion

CXCL12/CXCR4 ligand receptor interaction is involved in the directional migration of metastatic prostate cancer cells [34]. We found that SLUG positively regulates expression of the CXCL12/CXCR4 axis in human prostate cancer cell lines. Furthermore, we determined that forced expression of SLUG increased migration and invasion of human prostate cancer cells through activation of CXCR4/CXCL12 axis. Our findings add support that CXCL12 are a potential therapeutic target for prostate cancer metastasis [46].

## Materials and methods

### Cell Culture

PC3, 22RV1, LNCaP, and DU-145 cells were obtained from American Type Culture Collection (ATCC, Manassas, VA). These cells were maintained in culture medium, according to the manufacturer's instructions.

### Plasmids

pMig-Slug was constructed by cloning human SLUG gene into pMIGR1 retroviral vector. pLKO.1-Slug shRNA1 (target sequence: 5'-CAGCTGTAAATACTGTGACAA-3'), pLKO.1-Slug shRNA2 (target sequence: 5'- CCAAATCATTTCAACTGAAA-3'), pLKO.1-CXCL12 shRNA1 (target sequence: 5'-TGTGCATTGACCCGAAGCTAA), and pLKO.1-CXCL12 shRNA2 (target sequence: 5'-GCCAACGTCAAGCATCTCAAA-3') were obtained from Open Biosystem (Huntville, AL). pLKO.1 control shRNA (containing non-target scramble shRNA, Addgene plasmid #1864) were purchased from Addgene (Cambridge, MA).

### Viral Production and Infection

293T cells were seeded at 3 × 10^5 ^cells per well in a 6-well plate. The next day, a mixture of plasmid DNA was transfected separately into 293T cells using Superfect transfection reagent (Qiagen, Valencia, CA). For retrovirus production, pCL-Ampho (packaging plasmid) was mixed with pMig-based retroviral vectors. To generate the lentiviruses, the packaging plasmids (pCMV-VSVG and psPAX2) were co-transfected with pLKO.1-Slug shRNA or pLKO.1-control shRNA (containing non-target shRNA). The viruses were collected 24 hr after transfection. For viral infection, PC3, 22RV1, or DU-145 cells were seeded at 50% confluence in 6-well plates. The next day, the virus-containing supernatants from 293T cultures were mixed with polybrene (Sigma, St. Louis, MO) at a final concentration of 4 mg/ml, and added to the cells in each well. The plate was centrifuged at 2,000 rpm for 1 hr at 35°C, and returned to the cell culture incubator. PC3, 22RV1, DU145, and LNCaP cells were infected with retroviruses (pMig-Slug or pMig vector) for 3 times to achieve 100% transduction in these cells. Cells infected with pLKO.1 lentiviruses were selected with puromycin (1 μg/ml), starting at 48 hr after infection.

### RNA Isolation, cDNA Synthesis, RT-PCR, and qPCR Analysis

Total RNA extraction from cultured cells was accomplished by using RNeasy Plus mini kit (Qiagen, Valencia, CA). cDNA was synthesized by random priming from 1 μg of total RNA with the SuperScript III First-Strand Synthesis Super Mix kit (Invitrogen, San Diego, CA), according to the manufacturer's protocol. Primers used for the RT-PCR and qPCR analysis were synthesized by Integrated DNA Technologies (Coralville, IA). RT-PCR was performed by using the Hotstar Taq DNA polymerase kit (McLab, San Francisco, CA), and qPCR was performed by using the Perfecta SYBR Green FastMix (Quanta Bioscience, CA), according to the manufacturer's protocol. Data were analyzed by using the comparative CT method; CT refers to the ''threshold cycle,'' and is determined for each experiment using MyiQ software. Quantities of gene specific mRNA expression were determined by the CT method. Amplification of GAPDH was performed for each reverse-transcribed sample as an endogenous quantification standard. The fold-difference in gene expression was determined by 2^_ΔΔCT^. ΔΔCT is equal to (ΔC_T _of experimental conditions -ΔC_T _of control conditions). ΔC_T _is equal to (gene-specific C_T _-GAPDH C_T_). The primers are as following: *SLUG*, 5'-CTTCCTGGTCAAGAAGCA-3' and 5'-GGGAAATAATCACTGTATGTGTG-3'; *CXCR4*, 5'-ATATACACTTCAGATAACTACACCGAG-3' and 5'-TCAGTTTCTTCTGGTAACCCATGACCA-3'; CXCL12, 5'-ACCGCGCTCTGCCTCAGCGACGGGAAG-3' and 5' TGTTGTTCTTCAGCCGGGCTACAATCTG-3'; *MMP9*,5'-AGCGGGCGGCGCCTCTGGAGGTTCGA-3' and 5' CCTGGCAGAAATAGGCTTTCTCTCGGT-3'; *GAPDH*, 5' ATTGACCTCAACTACATGGTTTACATG-3' and 5'-TTGGAGGGATCTCGCTCCTGGAAG-3'.

### Enzyme-linked Immunosorbent Assay (ELISA)

Conditioned cell culture medium was centrifuged and an SDF1-α immunoassay kit (R&D Systems Inc. Minneapolis, MN) was used for CXCL12 detection. 100 μl of sample or control (or standard) was added into each well, according to the manufacturer's protocol. The optical density of each well was measured within 30 min, using a microplate reader set to 450 nm.

### Western Blot Analysis

The cells were lysed in the protein lysis buffer (20 mM Tris, 100 mM NaCl, 1 mM EDTA, 0.5% Triton X-100, 1 mM beta glycerophosphate, 1 mM sodium orthovanadate), supplemented with 1 ml protease inhibitor cocktail (Sigma, St. Louis, MO). The protein samples were analyzed by Western blot analysis using an ECL kit (Pierce, Rockford, IL) with antibodies against following antigens: Slug (ANASPEC, Fremont, CA), CXCR4 (Abcam, Boston, MA), GAPDH (Bethyl Laborotaries, Montgomery, TX).

### Zymographic Analysis of MMP activity

Cells overexpressing pMig or pMig-Slug (70-80% confluence) were washed twice with PBS, and the medium was changed to serum free cell culture medium. After 48 hr, the conditioned medium was collected and centrifuged for 5 min at 400 × g. A 500 μl aliquot was concentrated to < 100 ul in a Microcon concentrator (Millipore, Billerica, MA) at 6500 × g at 4°C. Protein concentration was determined using BCA assay (Thermo Scientific, Rockford, IL), and 20 μg of the protein from each sample was electrophoresed on a 10% zymography gel containing 0.1% gelatin (Invitrogen, San Diego, CA). MMP activity was detected by incubating the gel in 1× Zymogram Renaturing Buffer for 30 min at room temperature and then equilibrating the gel for 30 min at room temperature with gentle agitation. The gel was incubated with fresh 1× Zymogram Developing Buffer overnight, followed by staining with Coomassie Blue for 30 min. Contrast was adjusted by destaining with Coomassie destaining solution (Methanol: Acetic acid: Water (50: 10: 40). The staining gels were then air-dried in cellophane mounts and images were captured.

### Wound Healing Assay

The cells were seeded in a 12-well plate (15 × 10^4^). After the cells formed a confluent mono layer, scratches were performed using a 100 ul tip. The culture medium was replaced with fresh complete medium. The closure of scratch was analyzed under the microscope and images were captured at 18 - 24 hr after incubation.

### Invasion Assay

The cells were seeded at 6 × 10^4 ^cells per well into the 96-well plate of an Oris™ Cell Invasion Assay Kit (Platypus, Madison, WI). The plate was incubated for ~16 hr at 37°C. The stoppers were then removed. Collagen I Overlay was added to create a 3-D ECM environment for invasion and incubated for 1 hr at 37°C. Cell culture medium was added and the cells were allowed to invade for 72 hr, and were stained with DAPI before images were captured.

### Statistical Analysis

qPCR data and cell growth data were analyzed by the Student's *t*-test (one-tailed). P < 0.05 was used to define statistically significant differences.

## Competing interests

The authors declare that they have no competing interests.

## Authors' contributions

BU and WSW designed the experiments, participated in discussion of the data and draft of the manuscript. BU conducted experiments. All authors read and approved the final manuscript.

## Supplementary Material

Additional file 1 **Additional figure legend**. Figure legends for additional figures S1 - S4. **Figure S1 Flow Cytometric Analysis of CXCR4 expression on surface of LNCAP cells stably expressing SLUG**. SLUG overexpression (SLUG) and control (pMig) LNCAP cells were detached from plates with 4 mM EDTA solution when reaching 70-80% confluence. The cells were washed with 2% FBS in PBS solution and then stained with APC-labeled anti-human CXCR4 for flow cytometric analysis. **Figure S2 Analysis of MMP9 expression in PC3 cells stably expressing CXCL12 shRNAs**. RNA was extracted from PC3 infected with lentiviruses expressing CXCL12 shRNAs (sh1 and sh2), and used to synthesize cDNA. Transcript level of MMP9 and SLUG was analyzed by RT-PCR. GAPDH was included as a loading control. **Figure S3 Cell migration assay in 22RV1 cells stably overexpressing SLUG**. 22RV1 cells expressing pMig-Slug or pMig (vector) were seeded in 12-well plates (15 × 10^4 ^cells per well). After cells formed a confluent monolayer, scratches were performed using a 100 μl tip. Twenty-four hours after scratching, the cells were examined for closure of scratch under the microscope and images were captured. **Figure S4 Cell growth of PC3 stably expressing different combinations of CXCL12 shRNAs and SLUG**. Cell were seeded into 12 well plate (triplicates) at a density of 5 × 10^4 ^per well and viable cell numbers were counted with Beckman Vicell XR cell counter for 7 days. Click here for file

Additional file 2 **Additional figures**. Additional figures S1 - S4. Click here for file

## References

[B1] Jemal A Bray F Center MM Ferlay J Ward E Forman D Global cancer statistics CA Cancer J Clin 2011 61 69 90 10.3322/caac.20107 21296855

[B2] Liu J Uygur B Zhang Z Shao L Romero D Vary C Ding Q Wu WS Slug inhibits proliferation of human prostate cancer cells via downregulation of cyclin D1 expression Prostate 2010 70 1768 1777 20564361 10.1002/pros.21213 PMC2943978

[B3] Nieto MA The snail superfamily of zinc-finger transcription factors Nat Rev Mol Cell Biol 2002 3 155 166 11994736 10.1038/nrm757

[B4] Batlle E Sancho E Franci C Dominguez D Monfar M Baulida J Garcia De Herreros A The transcription factor snail is a repressor of E-cadherin gene expression in epithelial tumour cells Nat Cell Biol 2000 2 84 89 10.1038/35000034 10655587

[B5] Kudo-Saito C Shirako H Takeuchi T Kawakami Y Cancer metastasis is accelerated through immunosuppression during Snail-induced EMT of cancer cells Cancer Cell 2009 15 195 206 10.1016/j.ccr.2009.01.023 19249678

[B6] Hugo H Ackland ML Blick T Lawrence MG Clements JA Williams ED Thompson EW Epithelial--mesenchymal and mesenchymal--epithelial transitions in carcinoma progression J Cell Physiol 2007 213 374 383 10.1002/jcp.21223 17680632

[B7] Hajra KM Chen DY Fearon ER The SLUG zinc-finger protein represses E-cadherin in breast cancer Cancer Res 2002 62 1613 1618 11912130

[B8] Martin TA Goyal A Watkins G Jiang WG Expression of the transcription factors snail, slug, and twist and their clinical significance in human breast cancer Ann Surg Oncol 2005 12 488 496 10.1245/ASO.2005.04.010 15864483

[B9] Arai J Yasukawa M Yakushijin Y Miyazaki T Fujita S Stromal cells in lymph nodes attractB lymphoma cells via production ofstromal cell derived factor 1 European Journal of Haematology 2000 64 323 332 10.1034/j.1600-0609.2000.90147.x 10863978

[B10] Rossi D Zlotnik A The biology of chemokines and their receptors Annual review of immunology 2000 18 217 242 10.1146/annurev.immunol.18.1.217 10837058

[B11] Begley LA MacDonald JW Day ML Macoska JA CXCL12 activates a robust transcriptional response in human prostate epithelial cells J Biol Chem 2007 282 26767 26774 10.1074/jbc.M700440200 17631494

[B12] Marchesi F Monti P Leone BE Zerbi A Vecchi A Piemonti L Mantovani A Allavena P Increased survival, proliferation, and migration in metastatic human pancreatic tumor cells expressing functional CXCR4 Cancer Res 2004 64 8420 8427 10.1158/0008-5472.CAN-04-1343 15548713

[B13] Ma Q Jones D Borghesani PR Segal RA Nagasawa T Kishimoto T Bronson RT Springer TA Impaired B-lymphopoiesis, myelopoiesis, and derailed cerebellar neuron migration in CXCR4-and SDF-1-deficient mice Proceedings of the National Academy of Sciences of the United States of America 1998 95 9448 10.1073/pnas.95.16.9448 9689100 PMC21358

[B14] Balkwill F Cancer and the chemokine network Nature Reviews Cancer 2004 4 540 550 10.1038/nrc1388 15229479

[B15] Viola A Luster AD Chemokines and their receptors: drug targets in immunity and inflammation Annu Rev Pharmacol Toxicol 2008 48 171 197 10.1146/annurev.pharmtox.48.121806.154841 17883327

[B16] Teicher BA Fricker SP CXCL12 (SDF-1)/CXCR4 pathway in cancer Clinical Cancer Research 2010 16 2927 10.1158/1078-0432.CCR-09-2329 20484021

[B17] Eisenhardt A Frey U Tack M Rosskopf D Lummen G Rubben H Siffert W Expression analysis and potential functional role of the CXCR4 chemokine receptor in bladder cancer Eur Urol 2005 47 111 117 10.1016/j.eururo.2004.10.001 15582259

[B18] Cabioglu N Sahin AA Morandi P Meric-Bernstam F Islam R Lin HY Bucana CD Gonzalez-Angulo AM Hortobagyi GN Cristofanilli M Chemokine receptors in advanced breast cancer: differential expression in metastatic disease sites with diagnostic and therapeutic implications Ann Oncol 2009 20 1013 1019 10.1093/annonc/mdn740 19237480 PMC4318926

[B19] Tan CT Chu CY Lu YC Chang CC Lin BR Wu HH Liu HL Cha ST Prakash E Ko JY CXCL12/CXCR4 promotes laryngeal and hypopharyngeal squamous cell carcinoma metastasis through MMP-13-dependent invasion via the ERK1/2/AP-1 pathway Carcinogenesis 2008 29 1519 10.1093/carcin/bgn108 18487224

[B20] Scotton CJ Wilson JL Scott K Stamp G Wilbanks GD Fricker S Bridger G Balkwill FR Multiple actions of the chemokine CXCL12 on epithelial tumor cells in human ovarian cancer Cancer Res 2002 62 5930 5938 12384559

[B21] Pan J Mestas J Burdick MD Phillips RJ Thomas GV Reckamp K Belperio JA Strieter RM Stromal Derived Factor-1(SDF-1/CXCL 12) and CXCR 4 in renal cell carcinoma metastasis Molecular cancer 2006 5 56 10.1186/1476-4598-5-56 17083723 PMC1636662

[B22] Chinni SR Yamamoto H Dong Z Sabbota A Bonfil RD Cher ML CXCL12/CXCR4 transactivates HER2 in lipid rafts of prostate cancer cells and promotes growth of metastatic deposits in bone Mol Cancer Res 2008 6 446 457 10.1158/1541-7786.MCR-07-0117 18337451 PMC3842603

[B23] Gladson CL Welch DR New insights into the role of CXCR4 in prostate cancer metastasis Cancer biology & therapy 2008 7 1849 10.4161/cbt.7.11.7218 18981717 PMC2891934

[B24] Piva R Manferdini C Lambertini E Torreggiani E Penolazzi L Gambari R Pastore A Pelucchi S Gabusi E Piacentini A Slug contributes to the regulation of CXCL12 expression in human osteoblasts Experimental Cell Research 2010 317 1159 1168 21182836 10.1016/j.yexcr.2010.12.011

[B25] Zhang K Chen D Jiao X Zhang S Liu X Cao J Wu L Wang D Slug enhances invasion ability of pancreatic cancer cells through upregulation of matrix metalloproteinase-9 and actin cytoskeleton remodeling Laboratory Investigation 2011 91 426 438 10.1038/labinvest.2010.201 21283078 PMC3125102

[B26] Shanmugam MK Manu KA Ong TH Ramachandran L Surana R Bist P Lim LH Kumar AP Hui KM Sethi G Inhibition of CXCR4/CXCL12 signaling axis by ursolic acid leads to suppression of metastasis in transgenic adenocarcinoma of mouse prostate model Int J Cancer 2011 129 1552 1563 10.1002/ijc.26120 21480220

[B27] Sun YX Wang J Shelburne CE Lopatin DE Chinnaiyan AM Rubin MA Pienta KJ Taichman RS Expression of CXCR4 and CXCL12 (SDF-1) in human prostate cancers (PCa) in vivo J Cell Biochem 2003 89 462 473 10.1002/jcb.10522 12761880

[B28] Taichman RS Cooper C Keller ET Pienta KJ Taichman NS McCauley LK Use of the stromal cell-derived factor-1/CXCR4 pathway in prostate cancer metastasis to bone Cancer Res 2002 62 1832 1837 11912162

[B29] Tandon A Sinha S Structural insights into the binding of MMP9 inhibitors Bioinformation 5 310 314 10.6026/97320630005310 PMC3046033 21383916

[B30] Chinni SR Sivalogan S Dong Z Filho JC Deng X Bonfil RD Cher ML CXCL12/CXCR4 signaling activates Akt-1 and MMP9 expression in prostate cancer cells: the role of bone microenvironment-associated CXCL12 Prostate 2006 66 32 48 10.1002/pros.20318 16114056

[B31] Camp ER Findlay VJ Vaena SG Walsh J Lewin DN Turner DP Watson DK Slug Expression Enhances Tumor Formation in a Noninvasive Rectal Cancer Model J Surg Res 2011 170 56 63 10.1016/j.jss.2011.02.012 21470622 PMC3134530

[B32] Guleng B Tateishi K Ohta M Kanai F Jazag A Ijichi H Tanaka Y Washida M Morikane K Fukushima Y Blockade of the stromal cell-derived factor-1/CXCR4 axis attenuates in vivo tumor growth by inhibiting angiogenesis in a vascular endothelial growth factor-independent manner Cancer research 2005 65 5864 10.1158/0008-5472.CAN-04-3833 15994964

[B33] Darash-Yahana M Pikarsky E Abramovitch R Zeira E Pal B Karplus R Beider K Avniel S Kasem S Galun E Peled A Role of high expression levels of CXCR4 in tumor growth, vascularization, and metastasis FASEB J 2004 18 1240 1242 15180966 10.1096/fj.03-0935fje

[B34] Arya M Patel H McGURK C Tatoud R Klocker H Masters J Williamson M The importance of the CXCL12-CXCR4 chemokine ligand-receptor interaction in prostate cancer metastasis Journal of experimental therapeutics & oncology 2004 4 291 15844659

[B35] Xing Y Liu M Du Y Qu F Li Y Zhang Q Xiao Y Zhao J Zeng F Xiao C Tumor cell-specific blockade of CXCR4/SDF-1 interactions in prostate cancer cells by hTERT promoter induced CXCR4 knockdown: A possible metastasis preventing and minimizing approach Cancer biology & therapy 2008 7 1839 10.4161/cbt.7.11.6862 18836306

[B36] Frigo DE Sherk AB Wittmann BM Norris JD Wang Q Joseph JD Toner AP Brown M McDonnell DP Induction of Kruppel-like factor 5 expression by androgens results in increased CXCR4-dependent migration of prostate cancer cells in vitro Mol Endocrinol 2009 23 1385 1396 10.1210/me.2009-0010 19460858 PMC2737557

[B37] Pillai MM Yang X Balakrishnan I Bemis L Torok-Storb B MiR-886-3p down regulates CXCL12 (SDF1) expression in human marrow stromal cells PLoS One 5 e14304 10.1371/journal.pone.0014304 PMC3001477 21179442

[B38] Chu C Cha S Chang C Hsiao C Tan C Lu Y Jee S Kuo M Involvement of matrix metalloproteinase-13 in stromal-cell-derived factor 1 -directed invasion of human basal cell carcinoma cells Oncogene 2006 26 2491 2501 17099730 10.1038/sj.onc.1210040

[B39] Kukreja P Abdel-Mageed AB Mondal D Liu K Agrawal KC Up-regulation of CXCR4 Expression in PC3 Cells by Stromal-Derived Factor-1 (CXCL12) Increases Endothelial Adhesion and Transendothelial Migration: Role of MEK/ERK Signaling Pathway-Dependent NF- B Activation Cancer research 2005 65 9891 10.1158/0008-5472.CAN-05-1293 16267013

[B40] Thieme S Ryser M Gentsch M Navratiel K Brenner S Stiehler M Rolfing J Gelinsky M Rosen-Wolff A Stromal cell-derived factor-1alpha-directed chemoattraction of transiently CXCR4-overexpressing bone marrow stromal cells into functionalized three-dimensional biomimetic scaffolds Tissue Eng Part C Methods 2009 15 687 696 10.1089/ten.tec.2008.0556 19260802

[B41] Lin CY Tsai PH Kandaswami CC Lee PP Huang CJ Hwang JJ Lee MT Matrix metalloproteinase-9 cooperates with transcription factor Snail to induce epithelial-mesenchymal transition Cancer Sci 102 815 827 10.1111/j.1349-7006.2011.01861.x 21219539

[B42] Zhang K Zhang S Jiao X Wang H Zhang D Niu Z Shen Y Lv L Zhou Y Slug regulates proliferation and invasiveness of esophageal adenocarcinoma cells in vitro and in vivo Med Oncol 2010 in press 10.1007/s12032-010-9652-7 20730573

[B43] Casas E Kim J Bendesky A Ohno-Machado L Wolfe CJ Yang J Snail2 is an essential mediator of Twist1-induced epithelial mesenchymal transition and metastasis Cancer Res 71 245 254 10.1158/0008-5472.CAN-10-2330 PMC3025803 21199805

[B44] Shen X Wang S Wang H Liang M Xiao L Wang Z The role of SDF-1/CXCR4 axis in ovarian cancer metastasis Journal of Huazhong University of Science and Technology--Medical Sciences--2009 29 363 367 10.1007/s11596-009-0320-0 19513623

[B45] Towler D Cancer Interaction with the Bone Microenvironment American Journal of Pathology 2006 168 10.2353/ajpath.2006.050874 PMC1606588 16651608

[B46] Patrussi L Baldari CT The CXCL12/CXCR4 axis as a therapeutic target in cancer and HIV-1 infection Curr Med Chem 18 497 512 10.2174/092986711794480159 21143114

