# Supplementary material for: SLUG promotes prostate cancer cell migration and invasion via CXCR4/CXCL12 axis
Source: Mol Cancer. 2011 Nov 10;10:139. doi: 10.1186/1476-4598-10-139 (PMC3226635; doi:10.1186/1476-4598-10-139)
Supplement: Additional file 1 — Additional figure legend. Figure legends for additional figures S1 - S4. Figure S1 Flow Cytometric Analysis of CXCR4 expression on surface of LNCAP cells stably expressing SLUG. SLUG overexpression (SLUG) and control (pMig) LNCAP cells were detached from plates with 4 mM EDTA solution when reaching 70-80% confluence. The cells were washed with 2% FBS in PBS solution and then stained with APC-labeled anti-human CXCR4 for flow cytometric analysis. Figure S2 Analysis of MMP9 expression in PC3 cells stably expressing CXCL12 shRNAs. RNA was extracted from PC3 infected with lentiviruses expressing CXCL12 shRNAs (sh1 and sh2), and used to synthesize cDNA. Transcript level of MMP9 and SLUG was analyzed by RT-PCR. GAPDH was included as a loading control. Figure S3 Cell migration assay in 22RV1 cells stably overexpressing SLUG. 22RV1 cells expressing pMig-Slug or pMig (vector) were seeded in 12-well plates (15 × 104 cells per well). After cells formed a confluent monolayer, scratches were performed using a 100 μl tip. Twenty-four hours after scratching, the cells were examined for closure of scratch under the microscope and images were captured. Figure S4 Cell growth of PC3 stably expressing different combinations of CXCL12 shRNAs and SLUG. Cell were seeded into 12 well plate (triplicates) at a density of 5 × 10^4^ per well and viable cell numbers were counted with Beckman Vicell XR cell counter for 7 days. [file 1476-4598-10-139-S1.DOC]

**Additional files**

Additional figure legends

**Figure S1** **Flow Cytometric Analysis of CXCR4 expression on surface of LNCAP cells stably expressing SLUG**. SLUG overexpression (SLUG) and control (pMig) LNCAP cells were detached from plates with 4 mM EDTA solution when reaching 70-80% confluence. The cells were washed with 2% FBS in PBS solution and then stained with APC-labeled anti-human CXCR4 for flow cytometric analysis.

**Figure S2** **Analysis of MMP9 expression in PC3 cells stably expressing CXCL12 shRNAs**. RNA was extracted from PC3 infected with lentiviruses expressing CXCL12 shRNAs (sh1 and sh2), and used to synthesize cDNA. Transcript level of MMP9 and SLUG was analyzed by RT-PCR. GAPDH was included as a loading control.

**Figure S3** **Cell migration assay in 22RV1 cells stably overexpressing SLUG**. 22RV1 cells expressing pMig-Slug or pMig (vector) were seeded in 12-well plates (15 x 10^4^ cells per well). After cells formed a confluent monolayer, scratches were performed using a 100 μl tip. Twenty-four hours after scratching, the cells were examined for closure of scratch under the microscope and images were captured.

**Figure S4** **Cell growth of PC3 stably expressing different combinations of CXCL12 shRNAs and SLUG**. Cell were seeded into 12 well plate (triplicates) at a density of 5 x 10^4^ per well and viable cell numbers were counted with Beckman Vicell XR cell counter for 7 days.
