# Supplementary figures and images for: SLUG promotes prostate cancer cell migration and invasion via CXCR4/CXCL12 axis
Source: Mol Cancer. 2011 Nov 10;10:139. doi: 10.1186/1476-4598-10-139 (PMC3226635; doi:10.1186/1476-4598-10-139)

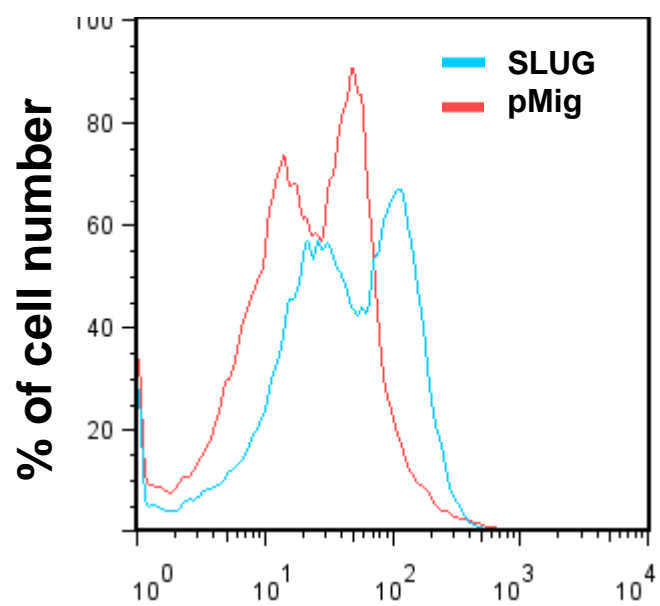

**Figure S1**

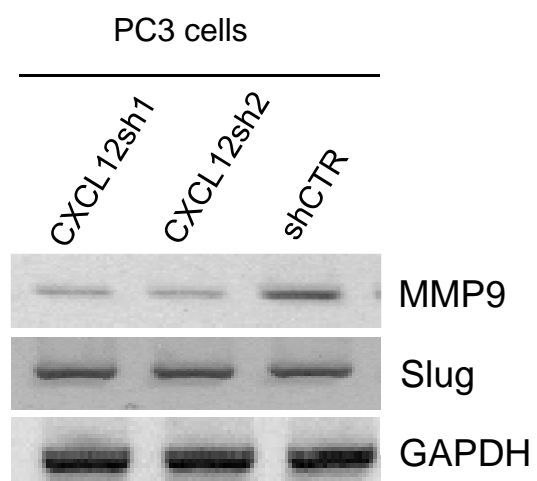

**Figure S2**

**A**

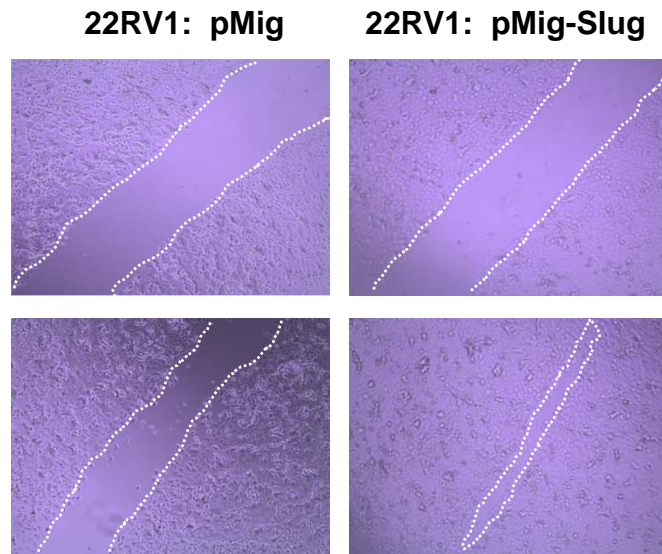

**B**

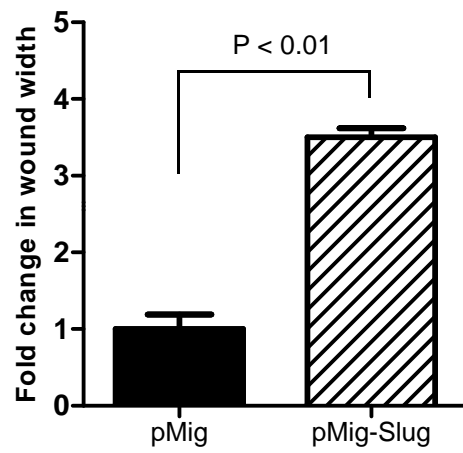

**Figure S3**

**A**

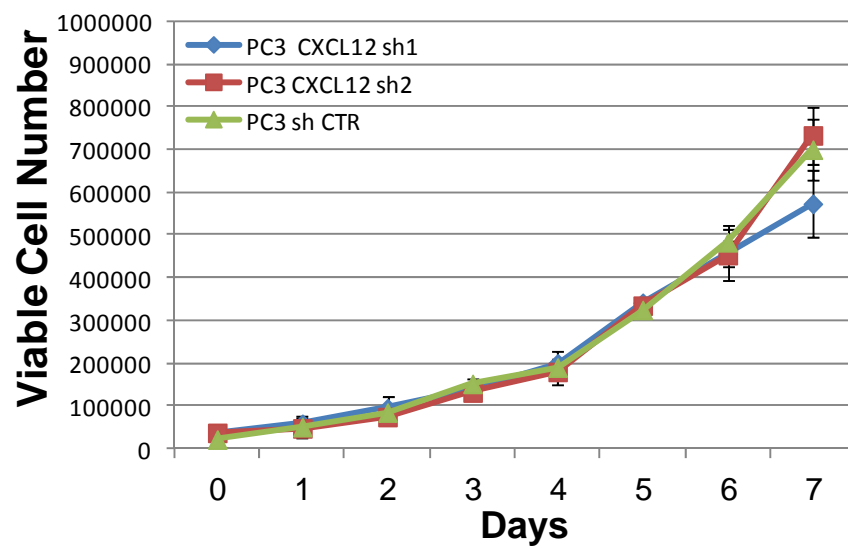

**B**

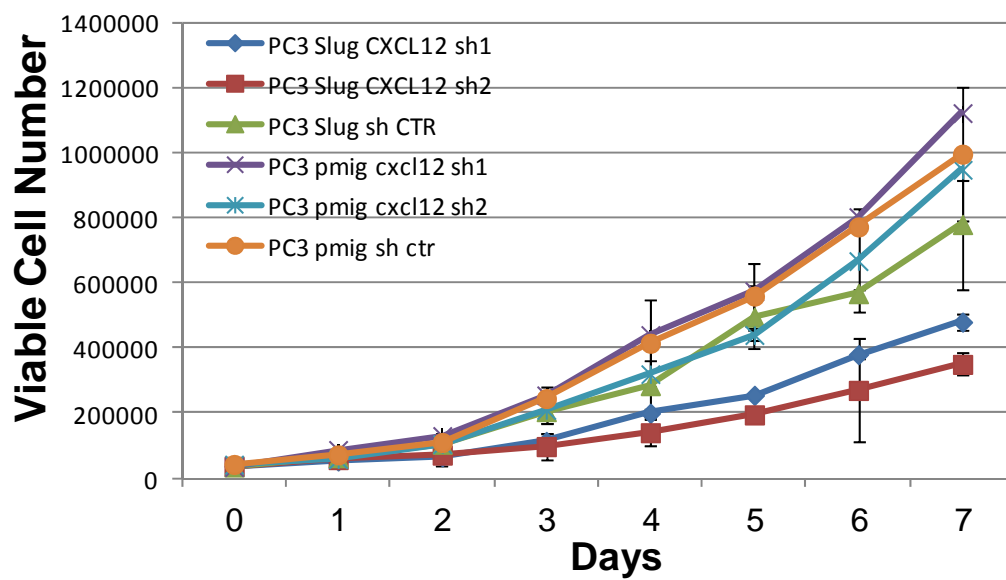

**Figure S4**

Supplement: Additional file 2 — Additional figures. Additional figures S1 - S4. [file 1476-4598-10-139-S2.PDF]
